# Inhaled Methane Limits the Mitochondrial Electron Transport Chain Dysfunction during Experimental Liver Ischemia-Reperfusion Injury

**DOI:** 10.1371/journal.pone.0146363

**Published:** 2016-01-07

**Authors:** Gerda Strifler, Eszter Tuboly, Edit Szél, Enikő Kaszonyi, Chun Cao, József Kaszaki, András Mészáros, Mihály Boros, Petra Hartmann

**Affiliations:** 1 Institute of Surgical Research, University of Szeged, Szeged, Hungary; 2 Department of Dermatology and Allergology, University of Szeged, Szeged, Hungary; Virginia Commonwealth University, UNITED STATES

## Abstract

**Background:**

Methanogenesis can indicate the fermentation activity of the gastrointestinal anaerobic flora. Methane also has a demonstrated anti-inflammatory potential. We hypothesized that enriched methane inhalation can influence the respiratory activity of the liver mitochondria after an ischemia-reperfusion (IR) challenge.

**Methods:**

The activity of oxidative phosphorylation system complexes was determined after *in vitro* methane treatment of intact liver mitochondria. Anesthetized Sprague-Dawley rats subjected to standardized 60-min warm hepatic ischemia inhaled normoxic air (n = 6) or normoxic air containing 2.2% methane, from 50 min of ischemia and throughout the 60-min reperfusion period (n = 6). Measurement data were compared with those on sham-operated animals (n = 6 each). Liver biopsy samples were subjected to high-resolution respirometry; whole-blood superoxide and hydrogen peroxide production was measured; hepatocyte apoptosis was detected with TUNEL staining and *in vivo* fluorescence laser scanning microscopy.

**Results:**

Significantly decreased complex II-linked basal respiration was found in the normoxic IR group at 55 min of ischemia and a lower respiratory capacity (~60%) and after 5 min of reperfusion. Methane inhalation preserved the maximal respiratory capacity at 55 min of ischemia and significantly improved the basal respiration during the first 30 min of reperfusion. The IR-induced cytochrome c activity, reactive oxygen species (ROS) production and hepatocyte apoptosis were also significantly reduced.

**Conclusions:**

The normoxic IR injury was accompanied by significant functional damage of the inner mitochondrial membrane, increased cytochrome c activity, enhanced ROS production and apoptosis. An elevated methane intake confers significant protection against mitochondrial dysfunction and reduces the oxidative damage of the hepatocytes.

## Introduction

The mitochondria integrate the oxidation of substrates with the reduction of molecular oxygen (O_2_) in the aerobic cell. A major threat to this equilibrium is hypoxia, when the lack of electron acceptor O_2_ leads to less ATP generation, and the accumulation of metabolic by-products. Re-establishment of the O_2_ flux is necessary but precarious, as the disturbed intracellular redox chemistry may lead to the formation of reactive oxygen species (ROS) with disturbances of the osmotic, ion and electric balances, structural membrane abnormalities and the activation of pro-death pathways.

In this system the availability of O_2_ is a vital issue, but it has become clear that other gaseous components of the cellular atmosphere are also of importance to the mitochondrial biology. Methane (CH_4_), a ubiquitous, small molecule, is a non-toxic, simple asphyxiant that can displace O_2_ in a restricted area. There is good reason to assume that this feature can influence the biology of eukaryote cells, though the role of CH_4_ in the mammalian physiology is largely unmapped and the effect of CH_4_ on mitochondrial homeostasis has never been investigated.

Mammalian methanogenesis is widely regarded as an indicator of the gastrointestinal (GI) carbohydrate fermentation by the anaerobic flora. Once generated by microbes or released by a nonbacterial process, CH_4_ is generally considered to be biologically inactive. However, some data do hint at an association with the small bowel motility regulation, as CH_4_ produced in the GI tract is usually associated with a decreased intestinal transit time, and other results suggest that CH_4_ production (usually defined as a > 1 ppm elevation of exhaled CH_4_ over the atmospheric level on breath testing) correlates with constipation in irritable bowel syndrome [1]. Information on the effects of exogenous CH_4_ is sparse, but a previous study demonstrated that CH_4_ supplementation can attenuate microcirculatory failure and the tissue accumulation of inflammatory cells in a large animal model of intestinal ischemia-reperfusion (IR) [2]. These data point to an anti-inflammatory potential for CH_4_, but the identification of intracellular targets remains elusive [2].

Liver diseases are often accompanied by mitochondrial functional disorders, and diseases of the mitochondria appear to cause damage to liver cells. On this basis, we set out to investigate the effects of increased CH_4_ inhalation on the function of the mitochondrial electron transport chain (ETC) in the liver of unstressed animals and after a standardized hypoxic insult. For this purpose, we employed a well-established IR model where the organ damage is mainly attributed to the enhanced activity of superoxide-generating enzymes and the failure of the mitochondrial ETC enzymes [3,4,5]. We postulated that, as they are critically involved in hypoxia-reoxygenation-induced intracellular respiratory damage, the mitochondria may be targets of CH_4_ administration. In particular, we hypothesized that, if CH_4_ is bioactive, it can exert its effect by influencing the respiratory activity and ROS production of the hepatic mitochondria.

## Materials and Methods

### *In vivo* experiments

The experiments were carried out on male Sprague-Dawley rats (average weight 300±20 g) housed in an environmentally controlled room with a 12-h light-dark cycle, and kept on commercial rat chow and tap water ad libitum. The experimental protocol was in accordance with EU directive 2010/63 for the protection of animals used for scientific purposes and was approved by the Animal Welfare Committee of the University of Szeged. This study also complied with the criteria of the US National Institutes of Health Guidelines for the Care and Use of Laboratory Animals.

### Surgical procedures

The rats were anesthetized with sodium pentobarbital (45 mg/kg ip), and the trachea was cannulated to facilitate respiration. The right jugular vein and carotid artery were cannulated for fluid and drug administration, respectively. Further small supplementary doses of pentobarbital were given iv when necessary. The animals were placed in a supine position on a heating pad to maintain the body temperature between 36 and 37°C, and Ringer's lactate was infused at a rate of 10 ml/kg/h during the experiments. For the preparation of the liver, the fur covering the abdomen was shaved, and the skin was disinfected with povidone iodide. After midline laparotomy and bilateral subcostal incisions, the liver was carefully mobilized from all ligamentous attachments; complete ischemia of the median and left hepatic lobes was achieved by clamping the left lateral branches of the hepatic artery and the portal vein with a microsurgical clip for 60 min. After the period of ischemia, the clips were removed and measurements were performed during a 60-min reperfusion period [5]. The wound was temporarily covered with non-water-permeable foil during the reperfusion period. At the end of experiments the animals were over-anesthetized with a single overdose of pentobarbital.

### Experimental protocols

**Fig 1:**
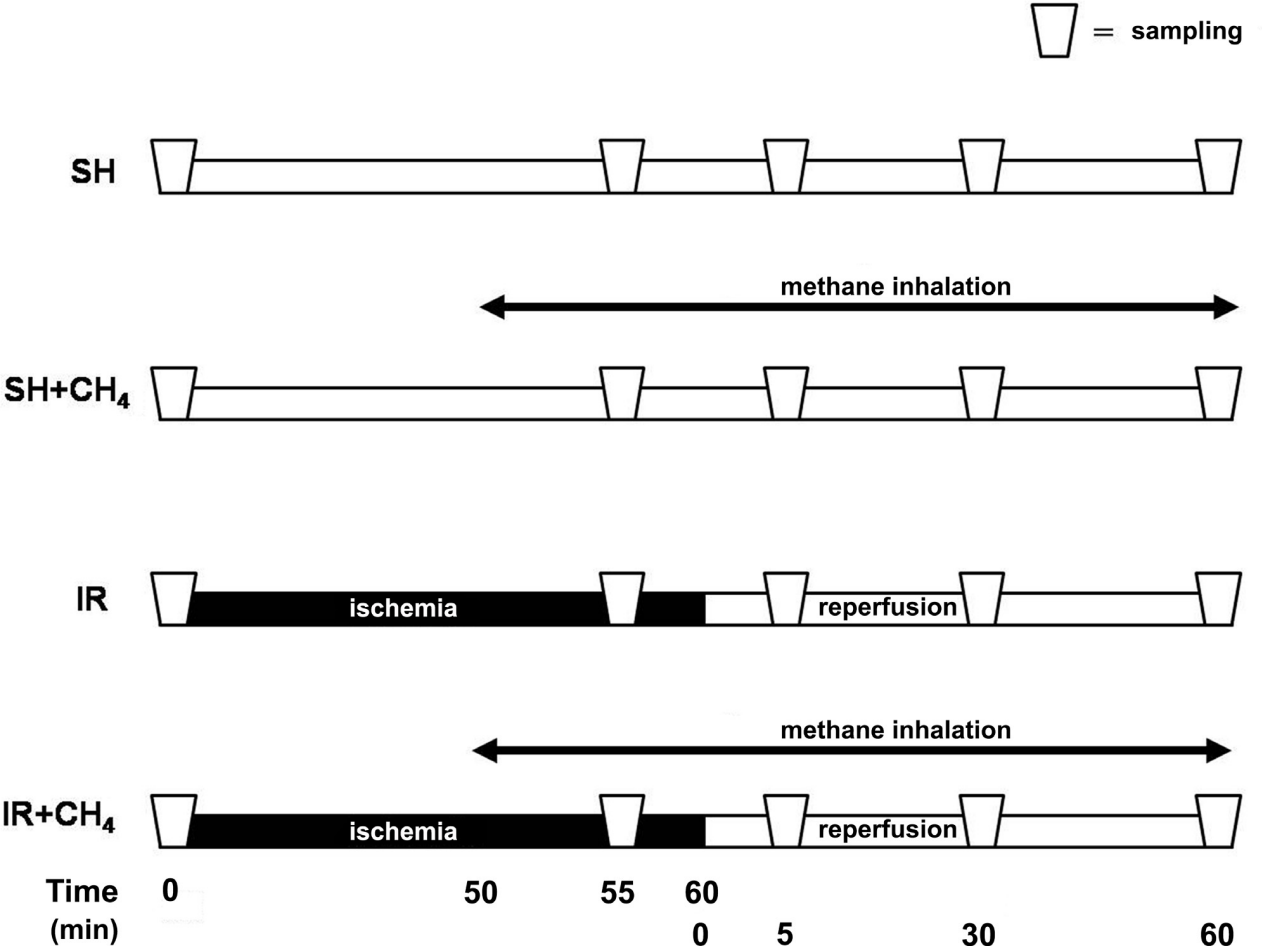
Experimental protocol. 60-min partial hepatic warm ischemia was induced with subsequent reperfusion (IR) in the presence or absence of 2.2% CH_4_ inhalation (IR and IR+CH_4_ groups). Sham-operated animals were subjected to the same surgical procedures, but without subsequent IR, and participated or not in 2.2% CH_4_ inhalation (SH+CH_4_ and SH groups). Liver samples for the analysis of the mitochondrial respiration were taken at baseline, at 55 min of ischemia, and at 5, 30 and 60 min of reperfusion.

The animals were randomly assigned to one or other of the following groups. In the IR group (n = 6), the mitochondrial respiratory functions in response to 60-min complete ischemia and 60-min reperfusion in normoxic air were examined. Control tissue samples were taken to determine the baseline mitochondrial respiratory variables, and ischemia was then induced in the median and left hepatic lobes by clamping the left lateral branches of the hepatic artery and the portal vein. At 55 min of ischemia, liver samples were taken for analysis of the mitochondrial respiration in response to ischemia. Following release of the vascular occlusions, biopsies were obtained from the affected lobes at 5 min, 30 and 60 min of reperfusion. In the IR+CH_4_ group (n = 6), the protocol was identical, except that inhalation with normoxic artificial air containing 2.2% CH_4_ (Linde Gas, Budapest, Hungary) was started after 50 min of ischemia and continued throughout the reperfusion period. The sham-operated animals in the SH group (n = 6) underwent the same surgical procedure but liver ischemia was not induced and the animals inhaled normoxic air, while the sham-operated animals in the SH+CH_4_ group (n = 6) were likewise not subjected to liver ischemia, but inhaled CH_4_ for the same duration as in the IR+CH_4_ (Fig 1).

Blood samples were taken for whole-blood superoxide, hydrogen peroxide (H_2_O_2_) and cytochrome c activity measurements at baseline and after 5, 15, 40 and 70 min of CH_4_ inhalation. Tissue biopsies for histological analysis (terminal deoxynucleotidyl transferase dUTP nick end labelling (TUNEL) and 4',6-diamidino-2-phenylindole (DAPI) staining) were taken at the end of the experiments and were stored at -80°C until assays.

Another series of animals (n = 3, each group) participated in identical treatment protocols and in vivo histology with laser-scanning confocal microscopy (LSCM) to determine the structural damage in the left liver lobe.

### Analysis of mitochondrial respiratory function

For determination of the respiratory activity in the liver mitochondria, tissue samples were homogenized in 3 ml MitOx2 (mitochondrial respiration medium) and then subjected to high-resolution respirometry with an Oxygraph-2k (Oroboros Instruments, Innsbruck, Austria). The steady-state basal O_2_ consumption (respiratory flux) was observed, and the complex II-linked state II respiration rate was then determined with 10 mM succinate after the addition of 0.5 μM complex I inhibitor rotenone. For determination of the maximum respiratory capacity of complex II-linked (state III respiration), 2.5 mM ADP was added to the chamber. Finally, the intactness of the outer mitochondrial membrane was assessed after 10 μM cytochrome c addition. The respirometry data were normalized to wet weight [6].

### Detection of cytochrome c oxidase activity

Cytochrome c oxidase activity was calculated via the time-dependent oxidation of cytochrome c at 550 nm, as described previously [7]. Briefly, a cytochrome c stock solution was freshly prepared by dissolving 10.6 mg cytochrome c (Sigma-Aldrich, Budapest, Hungary) in 20 ml distilled water. The cytochrome c was then reduced by adding 50 μl 0.1 M sodium dithionite, and the absorbance of the solution was determined at 550 nm; the photometer was calibrated to this level. Liver samples were homogenized with a Potter grinder in 10x ice-cold MitOx2 medium and then centrifuged at 800g for 5 min at 4°C. 50 μl supernatant was added to 2.5 ml cytochrome c stock solution and the decrease in optical density at 550 nm was measured spectrophotometrically during 1-min intervals at 0, 30 and 60 min.

### Malondialdehyde (MDA) assay on liver tissue

The lipid peroxide MDA level was measured through the reaction with thiobarbituric acid, by the method of Placer et al.[8] and corrected for the tissue protein content.

### Whole-blood superoxide and H_2_O_2_ production

10 μl whole-blood and 50 μl zymosan were added to 1ml Hank’s solution (PAA Cell Culture, Westborough, MA, USA) and the mixture was incubated at 37°C for 30 min, until assay [9]. The chemiluminometric response was measured with a Lumat LB9507 luminometer (Berthold Technologies, Wildbad, Germany) during a 30-min period after the addition of 100 μl of lucigenin and luminol reagent.

### LSCM and staining protocol

After mobilization of the liver from its ligamentous attachments, the left and median lobes were exteriorized, placed on a specially designed pedestal, and turned on the left side, providing a suitable horizontal plane of the liver lobe for examinations with the LSCM device. This method provided free access to the appropriate vessels while ensuring an adequate blood supply of the investigated liver lobes without the twisting of vascular pedicles. Confocal imaging with the LSCM device (FIVE1 system, Optiscan, Victoria, Australia) was started at the end of the reperfusion period. The rigid confocal probe (excitation wavelength 488 nm; emission detected at 505–585 nm) was mounted on a specially designed metal frame and gently pressed onto the liver surface [10].

For the in vivo staining of liver cells, 10 mg/ml fluorescein isothiocyanate (FITC)-labeled dextran (150 kDa) (Sigma-Aldrich, Budapest, Hungary) and 0.5 ml 0.01% acriflavine (Sigma-Aldrich, Budapest, Hungary) was injected into the jugular vein at the beginning of the reperfusion period.

### TUNEL and DAPI staining

Apoptosis was detected by the TUNEL method. For apoptotic cell staining, samples (n = 4–6) were analyzed with In situ cell death detection kit, TMR red (Roche, Cat. No 12 156 792 910) according to the manufacturer’s instructions. Briefly, tissue sections were fixed in 4% paraformaldehyde. For permeabilization, 0.1% Triton X-100 in 0.1% sodium citrate was used. The TUNEL reaction mixture comprised one part Enzyme Solution and nine parts Label Solution. Slides were incubated in a humidified atmosphere for 60 min at 37°C in the dark, followed by DAPI staining (Sigma-Aldrich®, 1:100). For each experimental series, one negative control (incubated with the Label Solution) and one positive control (digested with DNase I, grade I before application of the TUNEL reaction mixture) samples were used. Three pictures per sample were taken with a Zeiss AxioImager.Z1 microscope at 20x magnification. The number of apoptotic cells per field of view (524.19 μm x 524.19 μm) was determined by Image J 1.47 software.

### Statistical analysis

Data analysis was performed with SigmaStat statistical software (Jandel Corporation, San Rafael, CA, USA). Changes in variables within and between groups were analyzed by two-way repeated measures ANOVA, followed by the Bonferroni test in the cases of the mitochondrial respiratory function, cytochrome c activity from the mitochondria and whole-blood superoxide and H_2_O_2_ production; one-way ANOVA followed by the Holm-Sidak test was applied in the assay of MDA on liver tissue. Data were expressed as means ± SEM. For statistical analysis of TUNEL and DAPI staining, the Kruskal-Wallis and Dunn tests were applied. Histological data were expressed as median ± SD. Values of P < 0.05 were considered statistically significant.

### *In vitro* experiments

Pilot experiments were conducted to detect the changes in respiratory activity of different mitochondrial oxidative phosphorylation (OxPhos) system complexes in response to CH_4_ in intact mitochondria. For this purpose, liver mitochondria were isolated by the method of Gnaiger et al.[11]. Briefly, mitochondria were isolated from the left liver lobe in isotonic sucrose medium (300 mM sucrose, 0.2 mM EDTA and 10 mM HEPES, adjusted to pH 7.4 with KOH at 4°C). After the last centrifugation, mitochondrial pellets were resuspended in sucrose medium.

For respirometric analysis, isolated mitochondria were suspended in 3 ml MitOx2 medium and weighed into the chambers while the gas phase contained the 2.2% CH_4_-air mixture or room air (n = 8). The rate of respiration was determined after the addition of 2 mM malate and 10 mM glutamate for complex I-linked respiration, 2.5 mM ADP for complex I state III respiration, 10 mM succinate for complex I and complex II state III respiration, 0.5 μM rotenone for complex II-linked respiration and finally 2.5 μM antimycin A, 2 mM ascorbate and 20 μM N,N,N,N-tetramethyl-p-phenylenediamine (TMPD) for complex IV-linked respiration. DatLabTM software (Oroboros Instruments) was used for data analysis. The respirometry data were normalized to the mitochondrial biomass[6].

## Results

### I. Mitochondrial respiration and integrity

#### *In vivo* experiments

Respiratory activity in liver mitochondria.

**Fig 2:**
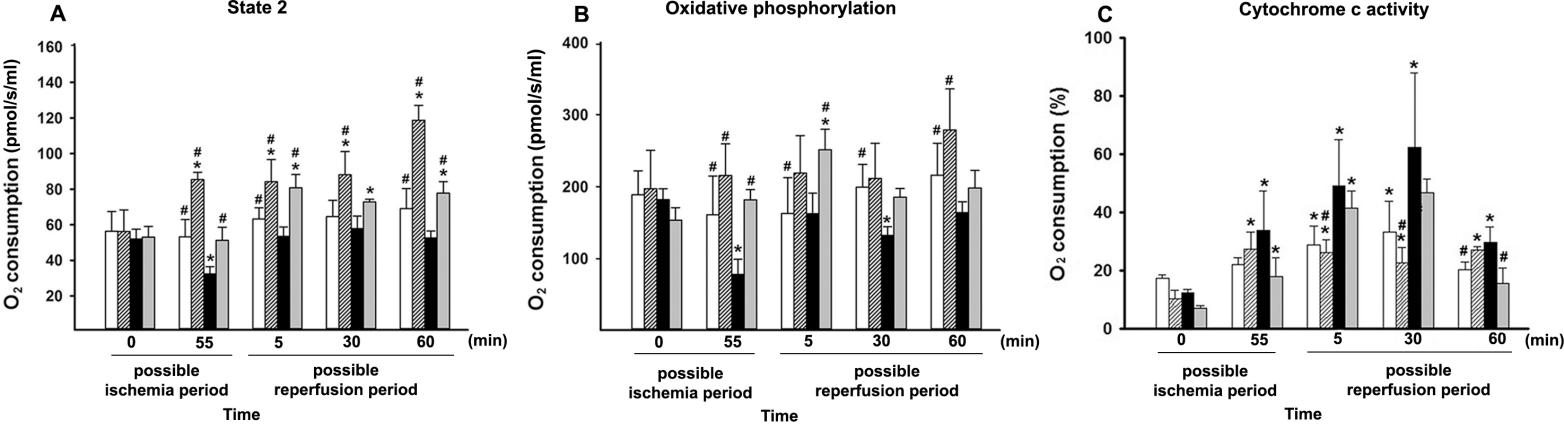
O_2_ consumption of liver homogenates measured by means of high-resolution respirometry. Animals were subjected to 60 min of liver ischemia followed by 60 min of reperfusion (IR group, black column) or were sham-operated (SH group, white column). 2.2% CH_4_ inhalation was started 10 min before the end of ischemia and continued throughout the reperfusion (IR+CH_4_ group, gray column), or during the identical interval in sham-operated animals (SH+CH_4_ group, striped column). (A) Basal respiration (complex II-coupled state II respiration) (in pmol/s/ml). (B) Oxidative phosphorylation (complex II-coupled state III respiration) (in pmol/s/ml). (C) Cytochrome c activity (state III respiration augmented by adding cytochrome c to the medium) (as % of state III respiration). Data are presented as means ± SEM. * P < 0.05 vs baseline; ^#^ P < 0.05 vs IR group (two-way ANOVA, Bonferroni test).

The efficacy of the liver mitochondrial ETC was assessed from a liver homogenate through high-resolution respirometry (Fig 2). The basal respiratory flux values (complex II-linked state II respiration) were significantly lower than those of the SH animals at 55 min of ischemia and at 60 min of reperfusion. The IR-induced decreases in basal flux were reversed in response to CH_4_ treatment. Interestingly, CH_4_ treatment alone (SH+CH_4_), elevated the basal O_2_ consumption throughout the observation period (Fig 2A).

In comparison with the SH group, IR resulted in a lower OxPhos capacity of the mitochondria (complex II-linked state III respiration) throughout the examination period. When CH_4_ inhalation was applied, however, the respiratory capacity was preserved at 55 min of ischemia and at 30 min of reperfusion (Fig 2B).

### Cytochrome c oxidase

**Fig 3:**
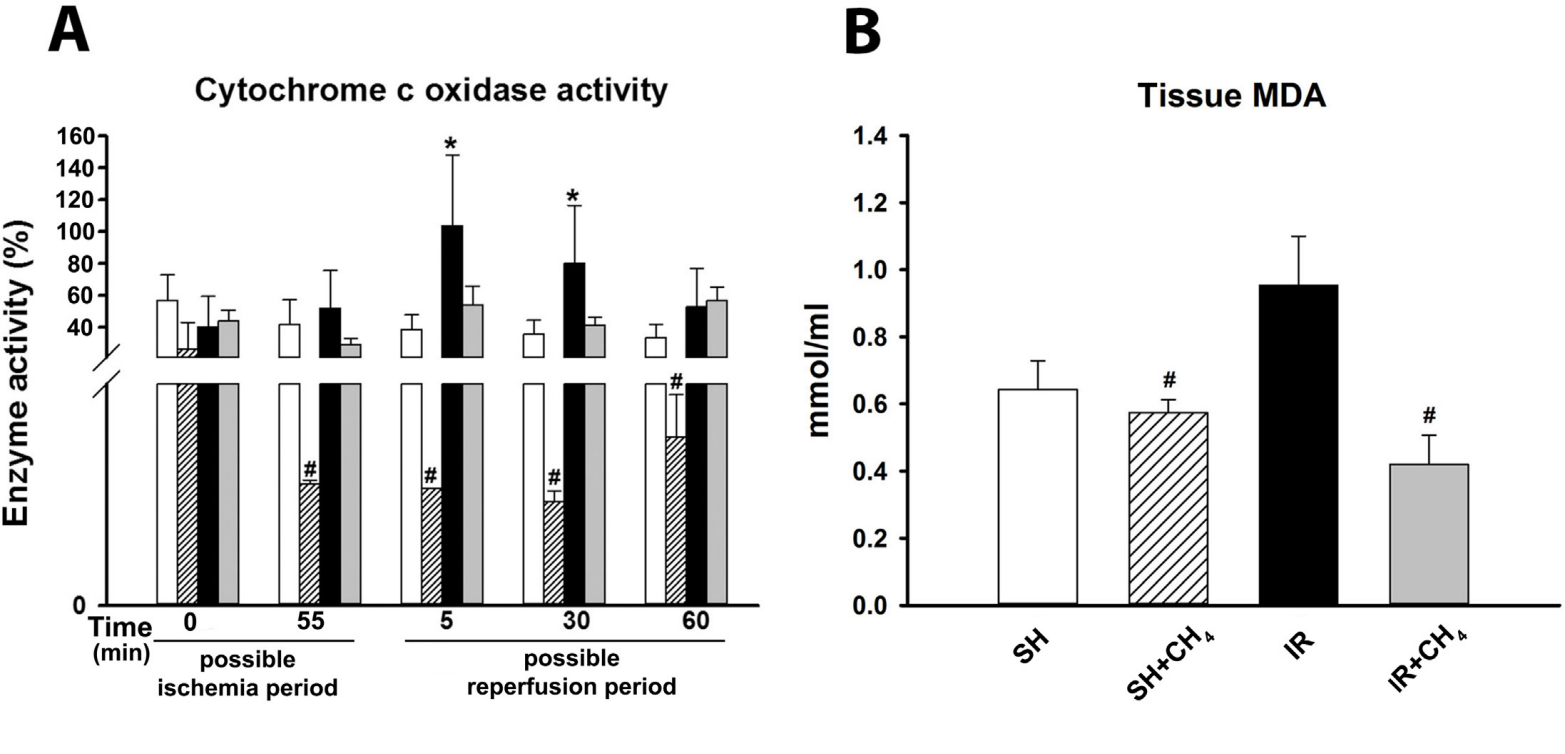
Cytochrome c activity and tissue MDA level in the rat liver. Animals were subjected to 60 min of liver ischemia followed by 60 min of reperfusion (IR group, black column) or were sham-operated (SH group, white column). 2.2% CH_4_ inhalation was started 10 min before the end of ischemia and continued throughout the reperfusion (IR+CH_4_ group, gray column), or during the identical interval in sham-operated animals (SH+CH_4_ group, striped column) (A) Cytochrome c activity (in %), (B) Tissue MDA level (in mmol/ml). Data are presented as means ± SEM. * P < 0.05 vs baseline; ^#^ P < 0.05 vs IR group (two-way ANOVA, Bonferroni test and one-way ANOVA, Holm-Sidak test).

The mitochondrial cytochrome c oxidase activity is an indicator of mitochondrial membrane damage. During our investigations, we determined the increase in mitochondrial respiration in response to exogenously administered cytochrome c by means of high-resolution respirometry (Fig 2C). The ability of exogenous cytochrome c to replace the enzyme in the inner mitochondrial membrane increased significantly as a result of the IR injury, while CH_4_ treatment restored the amount of exchanged enzyme to the baseline level. The cytochrome c oxidase activity was also determined with a spectrophotometric analysis (Fig 3A). In the SH animals, the cytochrome c level increased minimally as compared with the baseline during the experimental protocol. In the SH+CH_4_ group, the enzyme activity decreased in response to CH_4_ inhalation. In contrast, the IR group exhibited significantly higher cytochrome c oxidase activities during the reperfusion period, as an indication of functional damage. In the IR+CH_4_ group, the cytochrome c did not increase in response to the IR-induced damage (Fig 3A).

#### *In vitro* experiments

Basal mitochondrial respiration in intact mitochondria was achieved by adding substrates of complex I (Table 1). The saturating concentration of ADP resulted in a 2-fold increase in complex I-linked respiration, which was not affected by CH_4_ treatment. After a stable signal had been reached, complex II-dependent respiration was stimulated by adding succinate which caused a 6-fold increase in both groups. Complex I was then inhibited with rotenone to assess complex II-linked respiration. After complex III inhibition with antimycin-A, the residual O_2_ consumption was equal in the two groups. Finally, ascorbate and TMPD were added to the medium for the measurement of complex IV state III respiration; there was no significant difference in respiratory flux between the groups. Thus, incubation of the respiration medium with 2.2% CH_4_ did not affect the activity of OxPhos complexes as compared with room air (Table 1).

**Table 1:** Effects of CH_4_ incubation on O_2_ consumption (pmol/s/ml) of isolated intact liver mitochondria.

	Glutamate+Malate	ADP	Succinate	Rotenone	Antimycin A	Ascorbate+TMPD
**room air**	16,4±0,9	34,5±4,5	200,1±15,9	207,1±15,6	9,2±0,6	322,9±37,8
**2,2% CH**_**4**_	13,7±1,4	36,8±6,0	208,1±23,2	206,8±21,6	6,9±0,5	353,6±49,3

Data are presented as means ± SEM.

### II. Oxidative damage and lipid peroxidation products

#### Superoxide production

**Fig 4:**
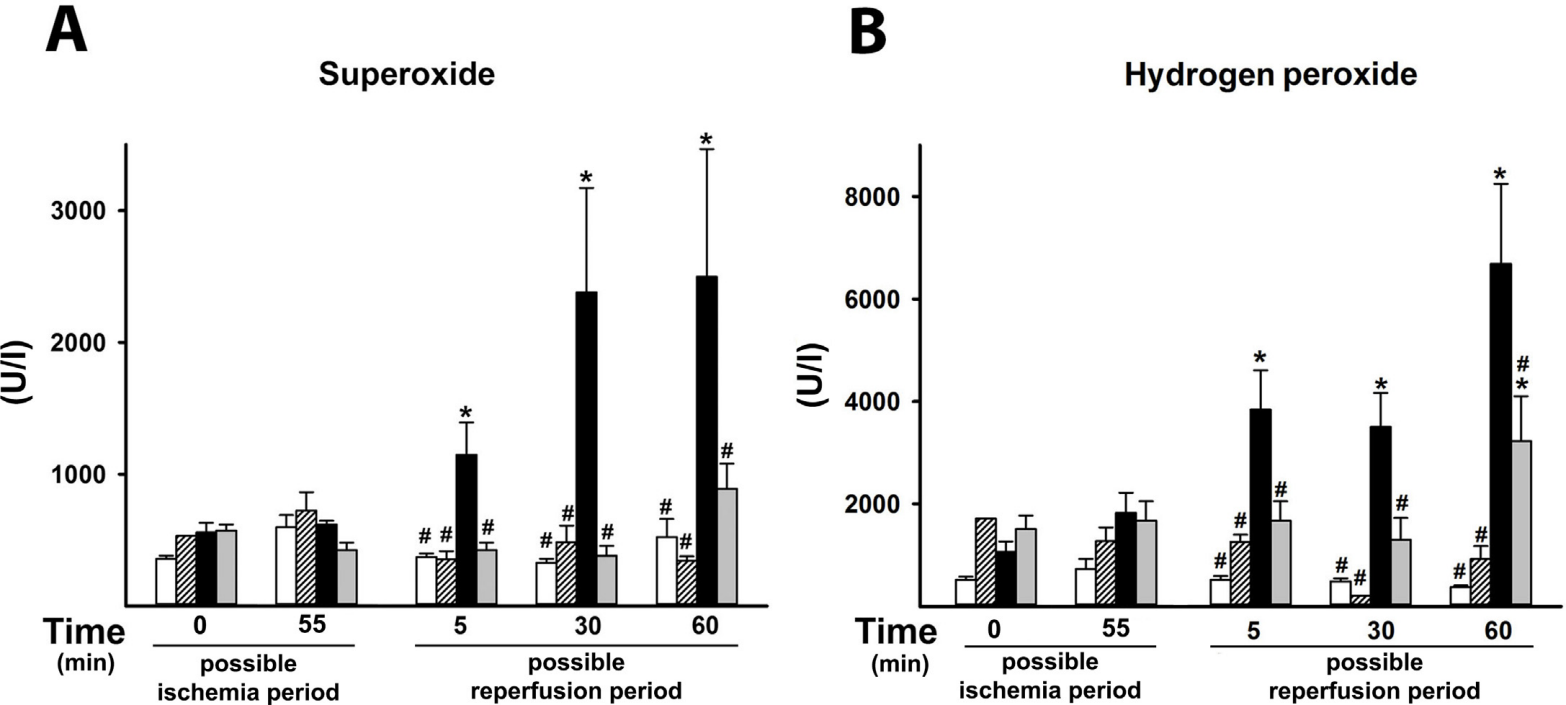
Superoxide and H_2_O_2_ production in the whole-blood. Animals were subjected to 60 min of liver ischemia followed by 60 min of reperfusion (IR group, black column) or were sham-operated (SH group, white column). 2.2% CH_4_ inhalation was started 10 min before the end of ischemia and continued throughout the reperfusion (IR+CH_4_ group, gray column), or during the identical interval in sham-operated animals (SH+CH_4_ group, striped column) (A) Superoxide level (in U/L), (B) H_2_O_2_ level (in U/L). Data are presented as means ± SEM. * P < 0.05 vs baseline; ^#^ P < 0.05 vs IR group (two-way ANOVA, Bonferroni test).

The whole-blood superoxide-producing capacity was significantly higher in the IR group at 30 min of reperfusion in comparison with the SH animals. The CH_4_ inhalation before the end of the ischemic period reduced the elevated superoxide production to the level in the SH animals (Fig 4A).

#### H_2_O_2_ production

Significantly higher whole-blood H_2_O_2_ levels were measured at 5, 30 and 60 min of reperfusion in the IR group relative to the SH group. The CH_4_ inhalation protocol effectively reversed H_2_O_2_ production in the IR+ CH_4_ group (Fig 4B).

#### MDA production

A significantly higher MDA level was measured at the end of reperfusion in the IR group than in the SH group. The IR-induced elevation of the liver MDA level was effectively attenuated by CH_4_ inhalation in the IR+CH_4_ group (Fig 3B).

### III. Structural damage

#### Apoptosis

**Fig 5:**
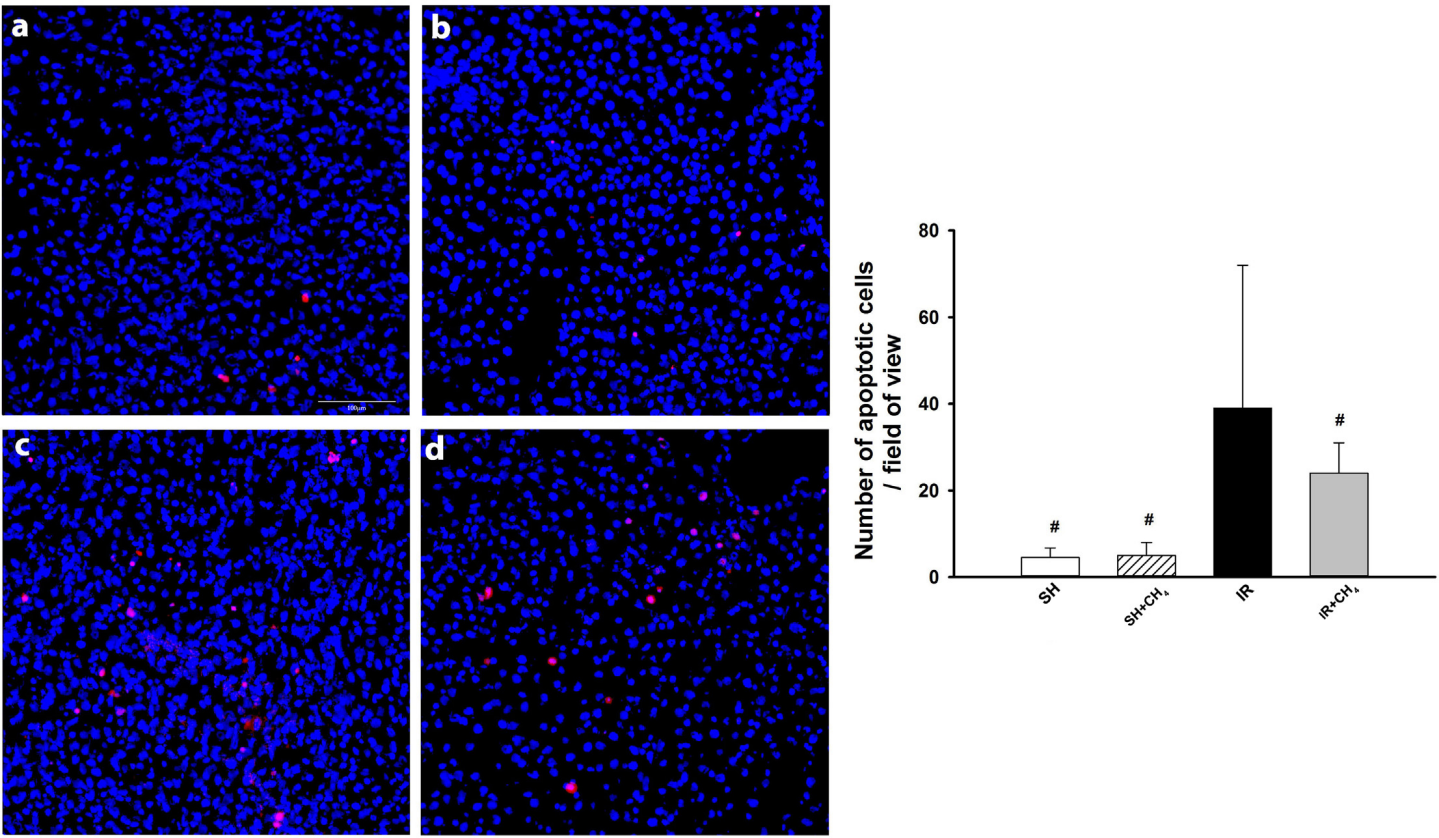
Liver cell apoptosis. Tissue sections are labeled with TUNEL/DAPI staining. (a) SH group, (b) SH+CH_4_ group, (c) IR group, (d) IR+CH_4_ group. Nuclei are marked in blue, and apoptotic nuclei in red. Data are presented as median ± SD. * P < 0.05 vs baseline; ^#^ P < 0.05 vs IR group (Kruskal-Wallis and Dunn tests).

As expected, few TUNEL-positive cells were observed in the liver specimens of the rats in the SH+CH_4_ and SH groups. Conversely, liver IR was accompanied by an increased TUNEL positivity, which was diminished as a result of CH_4_ inhalation (IR and IR+CH_4_ groups) (Fig 5).

#### *In vivo* morphological changes

**Fig 6:**
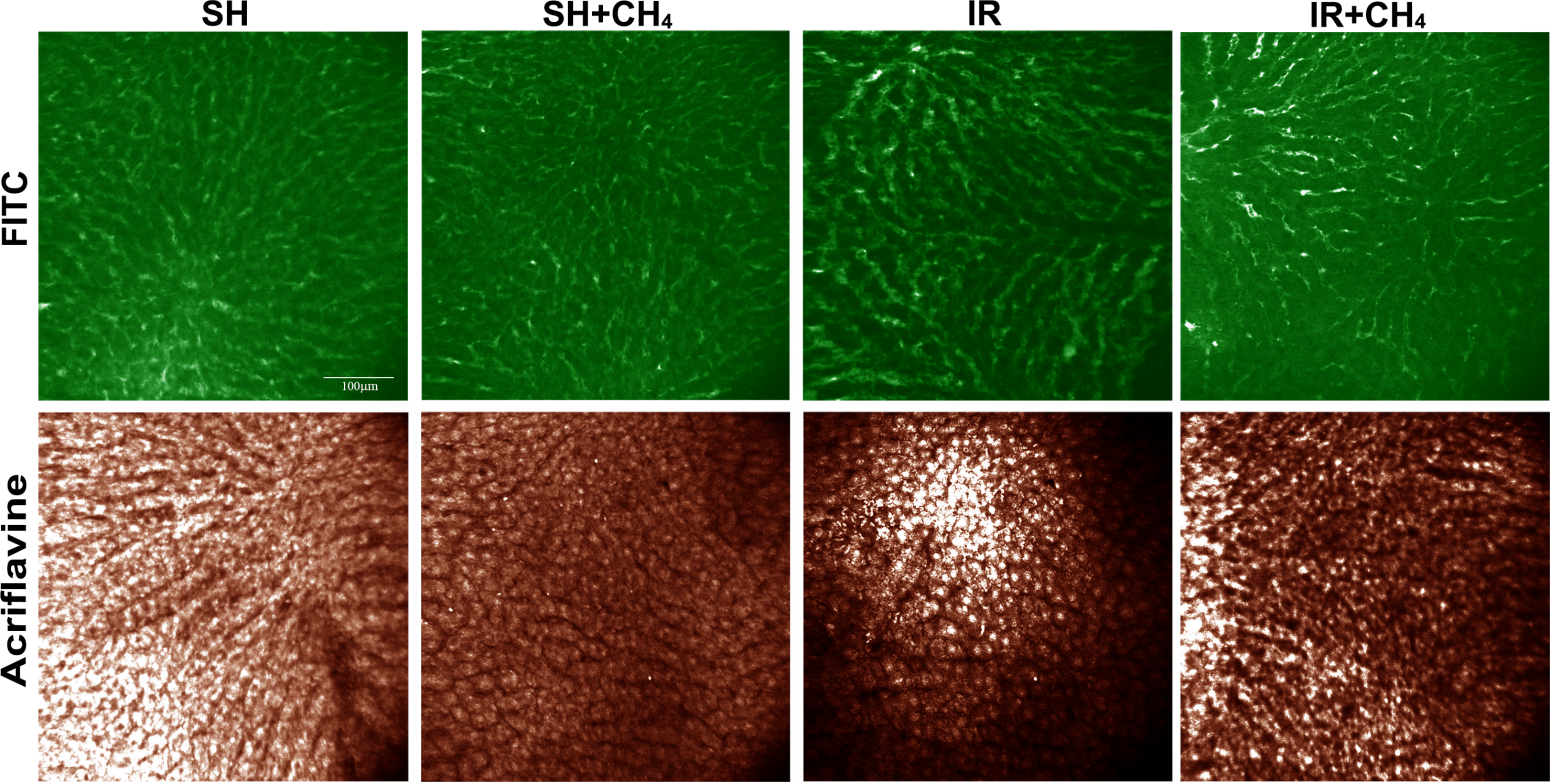
Histological changes in the rat liver. Tissue sections show the results of *in vivo* confocal laser scanning endomicroscopy with FITC dextran and acriflavine labeling. Apoptosis-related structural changes such as dilated sinusoids, loss of fluorescence intensity, changes in hexagonal cell shape, cytoplasmatic blebbing and vesicle formation can be observed in the IR group. The bar represents 100 μm.

The morphological changes in the left liver lobe were evaluated by means of in vivo imaging, using confocal laser scanning endomicroscopy. The FITC-dextran and acriflavine staining demonstrated more dilated sinusoids in the IR group, and also histological signs of apoptosis: a loss of fluorescence intensity, changes in hexagonal cell shape and cytoplasm blebbing and vesicle formation relative to the SH group. CH_4_ inhalation effectively attenuated these apoptosis-linked morphological changes (Fig 6).

## Discussion

A previous study has demonstrated the anti-inflammatory potential of CH_4_, but the identification of intracellular targets remains elusive. Accordingly, we investigated the in vivo influence of an increased CH_4_ intake on the respiratory activity of rat liver mitochondria by using controlled ventilation. This protocol did not influence the blood-gas chemistry in the anesthetized animals under the baseline conditions. More importantly, the inhalation of CH_4_-containing normoxic artificial air preserved the OxPhos after a period of tissue ischemia, and significantly improved the basal mitochondrial respiration state after the onset of reperfusion. In line with this, the IR-induced ROS production, cytochrome c activity and hepatocyte apoptosis were also reduced significantly.

In our experiments the non-phosphorylating basal respiration (state II respiration) of complex II in the presence of the reducing substrate succinate, but in the absence of ADP. The state II respiration is the resting state of the physiologically uncoupled or pathologically dyscoupled respiration, when the O_2_ flux is maintained in order to compensate for the proton leak at a high chemiosmotic potential, when ATP synthase is not active. ADP-dependent O_2_ consumption is a test of mitochondrial functional integrity and the overall function which directly reflects mitochondrial OxPhos [12]. The ADP-stimulated respiration of coupled mitochondria (OxPhos or state III) was supported by high, saturating concentrations of ADP and was significantly increased, by ~ 60%, in the SH animals. In contrast, significantly decreased complex II-linked basal respiration and a lower OxPhos capacity with higher superoxide and H_2_O_2_ production were found during reperfusion. The ADP-stimulated respiration was also decreased at the end of ischemia and during reperfusion.

The ROS production measured in this study can be linked to the mitochondria or to other sources, such as an increased activity of cellular oxidases. It is known that ~ 0,1–4% of the electrons flowing through the mitochondrial ETC generate superoxide anions by the partial reduction of O_2_ [13,14], i.e. involving precursors of ROS such as H_2_O_2_ and OH^.-^, and our data provide evidence of the influence of an increased CH_4_ intake on mitochondrial ETC reactions. However, the exact pathway through which CH_4_ influences the mitochondrial respiration remained unexplored and several mechanisms can be postulated to explain the in vivo effects of CH_4_ in this system. Interestingly, CH_4_ administration in sham animals elevated the state II O_2_ consumption throughout the observation period, which refers to an increased leak-dependent respiration. Lower mitochondrial ROS formation can partially be originated from the increased leak respiration in response to CH_4_ treatment.

In this respect, the data overall support a role for CH_4_ membrane defence during ROS-induced damage. During ischemia, the mitochondrial NADH/NAD^+^ and FADH/FAD^+^ ratios remain elevated, leading to reductive stress, [15,16] while reperfusion of the previously ischemic tissue leads to oxidative stress with a burst of ROS generation following the start of reoxygenation. It was earlier suggested (without indicating the exact biochemistry, contributing compounds or enzymes) that ROS reactions could lead to a higher level of fixation of CH_4_ in a lipid environment, such as the mitochondrium membrane [17,18]. Indeed, IR perturbs the heterogeneous lipid-bilayer membrane structure and changes the fluidity from fluid to gel. Disordered/fluidized bilayer states could therefore be analogous to physical damage to the ETC in these conditions. Inhaled exogenous CH_4_ will move from the alveoli into the circulation, and diffuse into the plasma, throughout which it is distributed rapidly and evenly. If there are no barriers, the apolar CH_4_ may enter the cytoplasm and mitochondrial matrix and dissolve in the hydrophobic nonpolar lipid tails of the phospholipid biomembranes [19]. Membrane rigidity relates to the degree of lipid peroxidation, when the level of oxidized lipids is increased and the fluidity of membranes is reduced. CH_4_ dissolved in biological membranes may affect this process, thereby influencing the stereo structure of membrane proteins, which determines their accessibility and morphology [20]. The peroxidation of polyunsaturated fatty acids and direct triggering of cytochrome c activity from the mitochondria are well-known consequences of IR injury [21]. The degree of lipid peroxidation can be estimated via the amount of MDA, a marker of oxidative damage of lipid membranes. As a reactive aldehyde, MDA is one of the many reactive electrophilic compounds that cause further oxidative stress in cells and form covalent protein adducts referred to as advanced lipoxidation end-products. We have shown that the levels of both ROS and MDA were reduced by an increased CH_4_ intake, indirectly demonstrating the decreased oxidative damage to the mitochondrial membranes.

Secondly, CH_4_ may accumulate transiently at cell membrane interfaces, thereby transitorily changing the physico-chemical properties or the in situ functionality of proteins, ion channels and receptors embedded within this environment. In this case, an increased CH_4_ intake may influence the function of membrane-bound structures. The pilot in vitro results demonstrated that the incubation of isolated mitochondria in a 2.2% CH_4_ atmosphere has no effect on the respiratory activity of the ETC. As opposed to this, the in vivo CH_4_ inhalation regimen unexpectedly increased the mitochondrial respiratory function, without influencing the baseline ROS production in the SH animals. These differing responses can also be explained by membrane injury, as compared to isolated mitochondria, homogenation can disrupt mitochondrial membranes [22]. In contrast, the protocol of mitochondria isolation permits rearrangement of the membranes and results in fully viable mitochondria whose function cannot be further ameliorated by CH_4_ [11].

This study addressed the possibility of CH_4_ bioactivity. This concept is supported by experimental data showing that gaseous CH_4_ can delay the contraction of peristalsis and increase the amplitude of the peristaltic contractions in the guinea pig ileum [23]. A recent study provided in vitro evidence that CH_4_ can inhibit the contractile activity of the smooth muscle by activating the voltage-dependent potassium channels and increasing the voltage-dependent potassium current [24].

Whereas the results indicate a bioactive role for higher concentrations of exogenous CH_4_, this is not obvious for endogenous sources. It is widely recognized that large amounts of CH_4_ can be produced by the anaerobic metabolism of methanogenic microorganisms in the GI tract [25,26], and CH_4_ is present in measurable amounts in the breath of approximately one-third of humans [27]. Nevertheless, as opposed to the previous view, in vitro and in vivo studies have revealed the possibility of nonmicrobial CH_4_ formation in both plants and animals [28,29]. CH_4_ generation has been demonstrated after site-specific inhibition of the ETC, and in association with a mitochondrial dysfunction, similarly to the effects of hypoxia [15,29].

Of interest, recent studies demonstrated the critical role of a ferryl species ([Fe(IV) = O]^2+^) and CH_3_ radicals, leading to the in vitro generation of CH_4_ from methionine sulfoxide as substrate at ambient temperature[30]. In this chemical reaction, CH_4_ is readily formed from the S-CH_3_ groups of organosulfur compounds in a model system containing iron(II/III), H_2_O_2_ and ascorbate that uses organic compounds with heterobonded CH_3_ groups for CH_4_ generation under ambient (1,000 mbar and 22°C) and aerobic (21% O_2_) conditions. CH_3_ radicals can be formed in the mitochondria through reaction between a reducing agent, a metal and a hydroperoxide. Methionine is known to be a key factor in many biochemical reactions in plants, fungi and animals, and methionine residues in the surface of proteins are highly susceptible to oxidation, the product generally being methionine sulfoxide. Importantly, the available data suggest that reversible methionine oxidation could be a novel mechanism in redox—regulation, which involves the repair mechanism of methionine sulfoxide reductases (MSRs) whose main function is to protect membranes from oxidative damage [20]. The MSRs were originally thought to be exclusively bacterial enzymes, but their presence was recently proven in mitochondria in mammals [31]. The scavenging action of these enzymes is based on the cyclic oxidation and reduction of the several methionine residues of the molecules that makes them the counterpart of the NADH/NAD^+^ system[20]. In a continuous lack of oxygen, the methionine sulfoxide-CH_4_ system may act as an alternative to NADH/NAD^+^ and FADH/FAD^+^ as an electron donor, thereby mitigating ischemia-induced reductive stress. The capacity of mitochondria to reverse oxidant-induced changes upon reperfusion originates from the exposure of previously hidden epitopes of mitochondrial proteins, as proven in the case of mitochondrial MSRs [32].

IR injury is an antigen-independent stimulus that initiates intrinsic signaling pathways. Peroxidation, an immediate chain reaction, causes a breakdown of biomembranes, leading to decompartmentalization, and the loss of maintenance of a steady state. Cytochrome c is attached to the inner membrane, and is detached in response to a threshold disturbance in the membrane structure, which leads to activation of the apoptotic caspase cascade [33]. CH_4_ inhalation effectively attenuated the IR-induced elevation in MDA level and in parallel the activity of cytochrome c was diminished. Conventional and in vivo histology provided evidence of IR-related apoptosis and the observations suggest that CH_4_ may influence the cell fate under stress conditions.

## Conclusions

Liver IR injury is a progressive process starting from a depressed mitochondrial ETC, the abnormal formation of ROS leading to biomembrane damage and finally necrotic or apoptotic cell death. Our experiments permit the conclusion that the mitochondrial protection afforded by CH_4_ inhalation involves different components under normal conditions, during ischemia and during reperfusion, similarly to the different pathomechanisms of damage during ischemia and after reperfusion. 2.2% CH_4_ inhalation clearly influenced the IR-related disturbances of the mitochondrial ETC, and mitigated the severity of subsequent events. The protective potential of CH_4_ was linked to reduced cytochrome c activity, and a reduced number of apoptotic hepatocytes. There is still no clear-cut evidence that CH_4_ in the endogenously produced concentration range (1–10 ppm) has a role in the cellular physiology, and further studies should be performed to determine whether endogenously-generated or exogenously-administered CH_4_ participates in restricting membrane fluidity and preserving the optimal condition of membrane-bound structures. Much remains unknown about hypoxic reactions and the fate of intracellular CH_4_ is an open question, but the results presented to date on the effects of CH_4_ against oxido-reductive biomembrane damage indicate a bioactive role for CH_4_.

## Supporting Information

S1 Table Effects of CH_4_ incubation on LEAK respiration (pmol/s/ml) in isolated intact liver mitochondria. Data are presented as means ± SEM.
(DOCX)
