# Supplementary material for: Inhaled Methane Limits the Mitochondrial Electron Transport Chain Dysfunction during Experimental Liver Ischemia-Reperfusion Injury
Source: PLoS One. 2016 Jan 7;11(1):e0146363. doi: 10.1371/journal.pone.0146363 (PMC4720186; doi:10.1371/journal.pone.0146363)
Supplement: S1 Table — Data are presented as means ± SEM. (DOCX) [file pone.0146363.s001.docx]

We have performed these additional *in vitro* experiments with isolated mitochondria in order to measure the maximal leak-dependent respiration.

Briefly, isolated mitochondria were suspended in 3 ml MitOx2 medium and placed into the chambers, while the gas phase contained the 2.2% CH_4_-air mixture or room air (n=6). The complex II-linked state II respiration rate was then determined with 10 mM succinate after the addition of 0.5 μM complex I inhibitor rotenone. For determination of the complex II-linked state III respiration, 2.5 mM ADP was added to the chamber. Finally, 5 mM oligomycin was added to the medium. The respiratory control ratio was calculated by dividing LEAK by the state II respiration of the mitochondria. The results are summarized below. In brief, the leak respiration was 125 ± 4 and 127 ± 5 pmol/s/ml in the room air and the 2.2% CH_4_-air mixture groups of isolated mitochondria; hence, these novel data do not indicate any effect of CH_4_ on intact mitochondria under *in vitro* circumstances. (The explanation for this phenomenon is discussed in detail in para 2, on page 15. Nonetheless, these *in vitro* data cannot exclude a role of the increased leak respiration in *in vivo* experiments involving a lower ROS production.

|  | **Room air** | **2.2% CH_4_-air** |
| --- | --- | --- |
| Rotenone / Succinate | 157 ± 6 | 155 ± 6 |
| OxPhos | 684 ± 39 | 607 ± 28 |
| LEAK oligomycin | 125 ± 4 | 127 ± 5 |
| Respiratory control ratio (RCR) | 0.79 | 0.82 |

**S1 Table.** Effects of CH_4_ incubation on LEAK respiration (pmol/s/ml) in isolated intact liver mitochondria. Data are presented as means ± SEM.
